# Supplementary material for: Neural correlates of evidence accumulation during value-based decisions revealed via simultaneous EEG-fMRI
Source: Nat Commun. 2017 Jun 9;8:15808. doi: 10.1038/ncomms15808 (PMC5472767; doi:10.1038/ncomms15808)
Supplement: Supplementary Information [file ncomms15808-s1.pdf]

Type of file: pdf
Size of file: 0 KB
Title of file for HTML: Supplementary Information
Description: Supplementary Figures, Supplementary Tables, Supplementary Methods and Supplementary References

Type of file: pdf
Size of file: 0 KB
Title of file for HTML: Peer Review File
Description:

SUPPLEMENTARY FIGURES

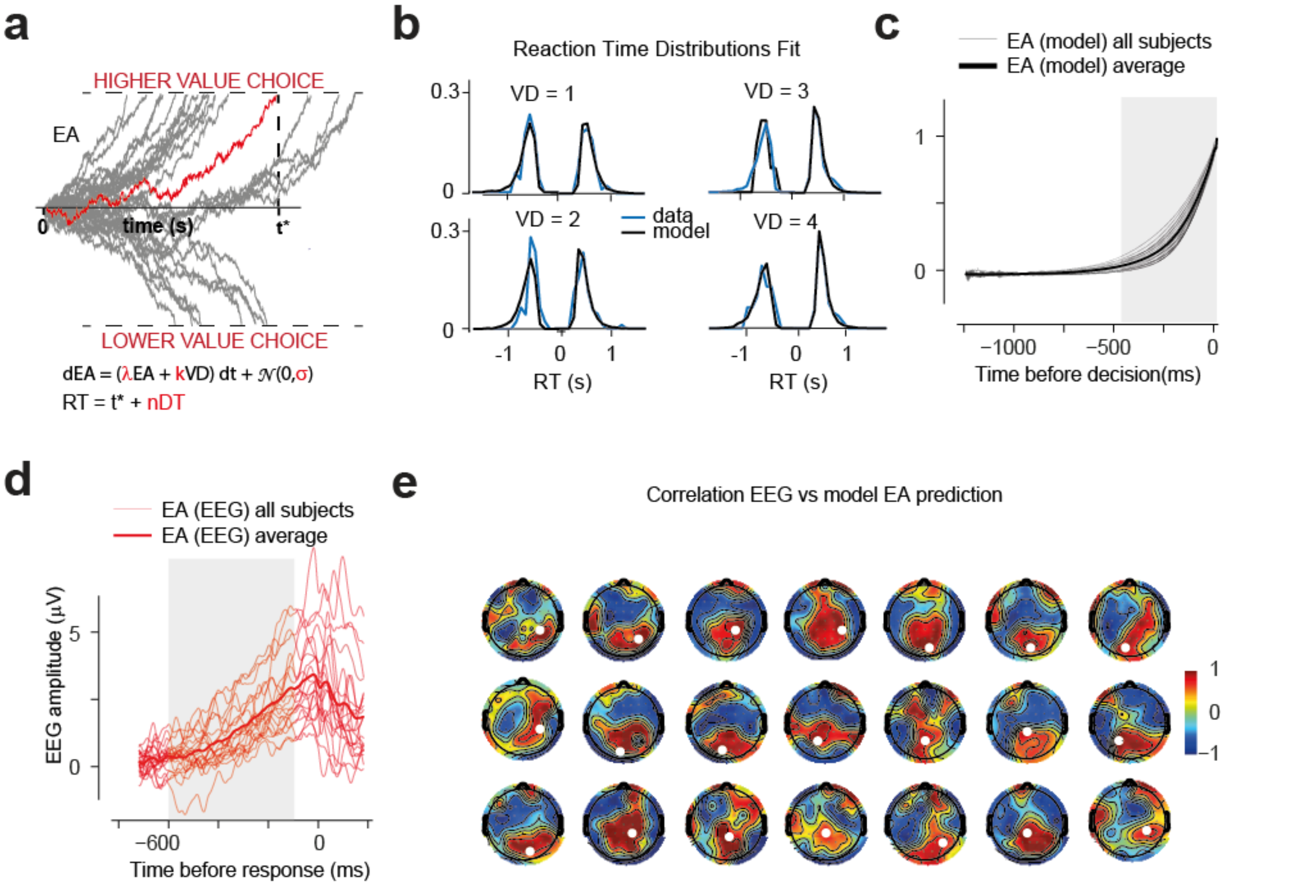

Supplementary Figure 1. Modeling of behavior and EEG-derived representation of Evidence Accumulation (EA). **(a)** Schematic representation of the Ornstein-Uhlenbeck process. In each trial EA of the value difference (VD) between the alternatives evolves over time according to the first equation on the bottom (see Methods in the main text for details). The decision time (t*) is established when the EA signal reaches a threshold (+1 when the model choose the higher value item (correct decision), -1 otherwise). Reaction Times (RTs) are obtained by adding a non-decision time (nDT) to the decision time of individual trials (second equation on the bottom). **(b)** Model fits of actual reaction time distributions for a representative subject for each of the VD levels for “correct” and “error” choices. Error trial RTs have been flipped in sign to combine them in a single distribution with the correct ones. **(c)** Model-derived EA predictions for all subjects (thin gray traces) and population average (thick black trace) obtained by averaging together all simulated trials for the best set of parameters of each subject. The shaded area depicts the time interval we considered for the

correlation with the EEG (see methods). **(d)** EEG-derived EA predictions for all subjects (thin traces) and population average (thick trace) obtained by averaging together all trials for the subject-specific electrode exhibiting the highest correlation with the model's prediction. The shaded area depicts the time interval we considered for correlation with the model-derived predictions (600 to 100 ms prior to the response) during which the build-up of evidence accumulation unfolds (see Methods section in the main text). **(e)** Individual subject scalp topographies of the relationship (correlation) between response-triggered EEG traces and EA predictions produced by the model. The white dots show the subject-specific electrodes that were most predictive of the model-derived EA over time. Note the consistency in this positive relationship across participants in midline centroparietal electrode locations.

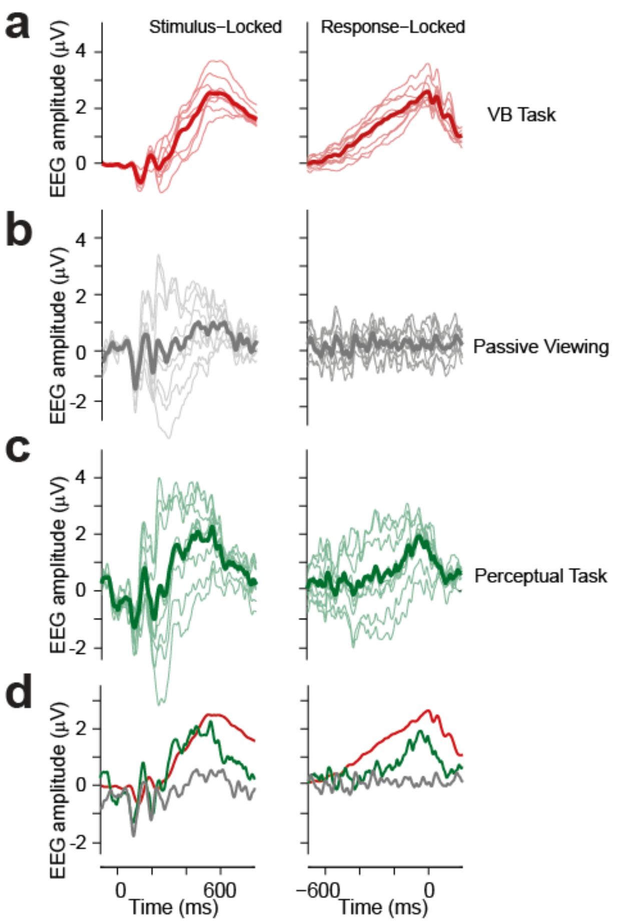

Supplementary Figure 2. Accumulation activity during value-based decisions, passive viewing and perceptual decisions (see Supplementary Methods)**.** (**a**) Population average (N = 21) stimulus- (left) and response-locked (right) EEG activity during the value-based (VB) task from all electrodes in the centroparietal cluster defined in Figure 1c. All 9 electrodes (thin traces) and the cluster average (thick trace) show accumulating activity. **(b)** Population average (N = 8) stimulus- and response-locked EEG activity during passive viewing of pairs of the same stimuli used for the VB task. The response-locked trace is obtained assuming subjects responded with the same mean RT as in the perceptual task and is used only for illustration purposes. All 9 electrodes (thin traces) and the cluster average (thick trace) show a perceptual response to the stimuli (within the first 200-250 ms) but no accumulating activity later on. **(c)** Population average (N = 8) stimulus- and response-locked EEG activity during a perceptual decision making task. Participants were presented with pairs of the same stimuli used for the VB task and asked to decide which items was larger in size. All 9 electrodes (thin traces) and the cluster average (thick trace) show both an early perceptual

response and a later accumulating activity. **(d)** Stimulus- and response-locked population averages for the VB (red), perceptual (green) and passive viewing (grey) tasks. The accumulation activity persists longer for the VB task compared to the perceptual task, consistent with longer integration/response times (<RT> = 772 ± 20 ms for VB task, <RT> = 505 ± 130 ms for perceptual decision making).

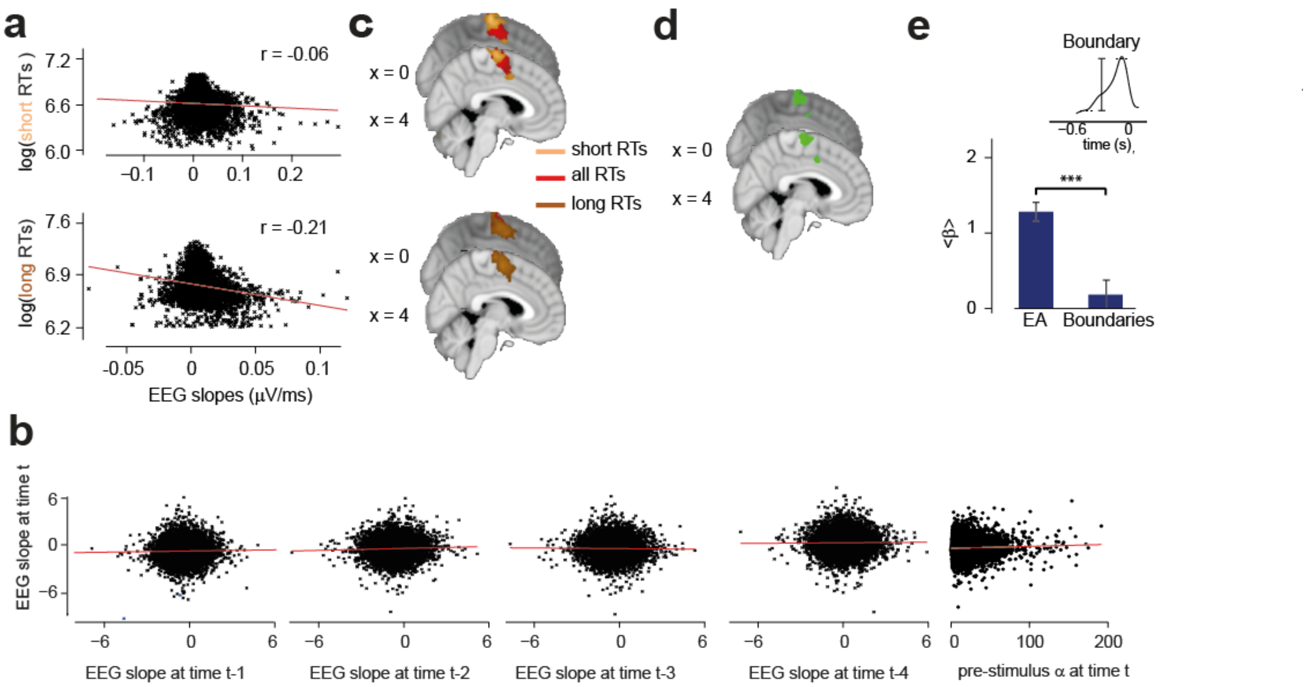

Supplementary Figure 3. pMFC correlated with evidence accumulation both in trials with short or long RTs. **(a)** Relationship between linear slopes of the single-trial response-triggered EEG traces and short (top plot) or long (bottom plot) RTs. For each participant, RTs were grouped into two bins through a median split. (**b**) Scatter plots of z-scored EEG slopes at trial t against EEG slopes at a number of immediately preceding trials and against pre-stimulus α power in the same trial. For each subject we ran a linear serial autoregression model predicting the slope in the current trial from the slopes from the past four trials. On average over the population, this model explained only a minimal fraction of the variance in the EEG slopes ($\langle R^2 \rangle = 0.019$). Increasing the number of past trials (up to six) in the autoregression model did not change the amount of explained variance. Similarly, we ran a linear regression model to predict the slopes of EA in each trial using the pre-stimulus α power as an index of attention in the same trial [1]. Pre-stimulus power in the α frequency band (8-12 Hz) was obtained using a wavelet transform as in [2]. In short, we convolved single trials using a complex Morlet wavelet. Single-trial power was calculated by averaging the squared absolute values of the convolutions in the 500 ms preceding the onset of the stimulus. For each subject we considered the peak α frequency in the occipitoparietal sensor with the highest overall α power. On average, this model explained only a minimal fraction of the variance in the EEG slopes

($\langle R^2 \rangle = 0.008$). Taken together these results indicate that local fluctuations in attention could not reliably explain the trial-by-trial changes in the slope of the EEG accumulating activity. For illustration each plot here comprises all trials from all subjects. **(c)** Separate EEG-informed fMRI predictors for short (top panel) and long (bottom panel) RT trials revealed activity in the same region of the pMFC seen in the main analysis (all RTs). **(d)** Similarly, a conjunction analysis between the two groups of trials revealed a significant overlap in activity in the same area. (**e**) Average β regression coefficients in pMFC (N=21) for the EEG regressor representing the full temporal dynamics of the decision process (within trial-specific time windows – see methods) and for a parametric regressor modulated by single-trial decision boundaries estimated from the EEG ('boundary' regressor). Specifically, these boundary estimates were computed as the difference between the signal amplitude at the onset and offset of the accumulation, (averaged within a 50 ms window centered at these times) (inset). The EEG regressor was a significantly better predictor of the fMRI signal in the pMFC than the 'boundary' regressor ($t(20) = 4.21$, $p < 0.001$; paired *t*-test comparison of the β coefficients in pMFC).

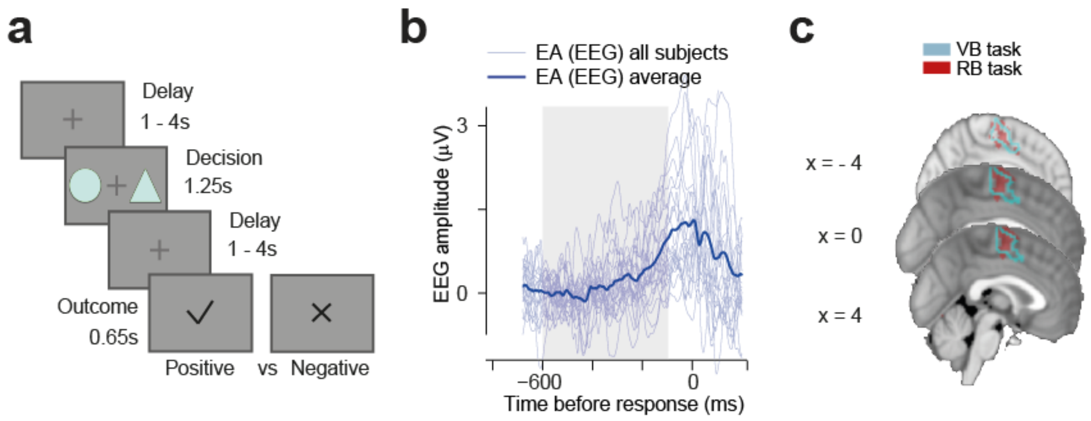

Supplementary Figure 4. Task design, EEG and EEG-informed fMRI in the probabilistic reward-based (RB) task (see Supplementary Methods). **(a)** On each trial, subjects had up to 1.25s to choose which of two abstract symbols was more likely to lead to a reward. After a random delay (1-4s) following their decision, subjects were informed about either a positive outcome (a tick) or a negative (non-rewarding) outcome (a cross). The same twenty-one participants as in the value-based (VB) task performed 2 blocks of 170 trials each. **(b)** EEG-derived EA predictions for all subjects (thin traces) and population average (thick trace) obtained by averaging together all trials for the "best" electrode of each subject obtained from the VB task (i.e. we treated the RB as a test dataset against which to validate the results of the VB task). The shaded area depicts the time interval when evidence accumulation occurs. **(c)** We repeated the same EEG-informed fMRI analysis as in the VB task using individual EA EEG traces from the RB task and identified a significant overlap in activity in pMFC across the two tasks (VB: red cluster outline; RB: blue cluster).

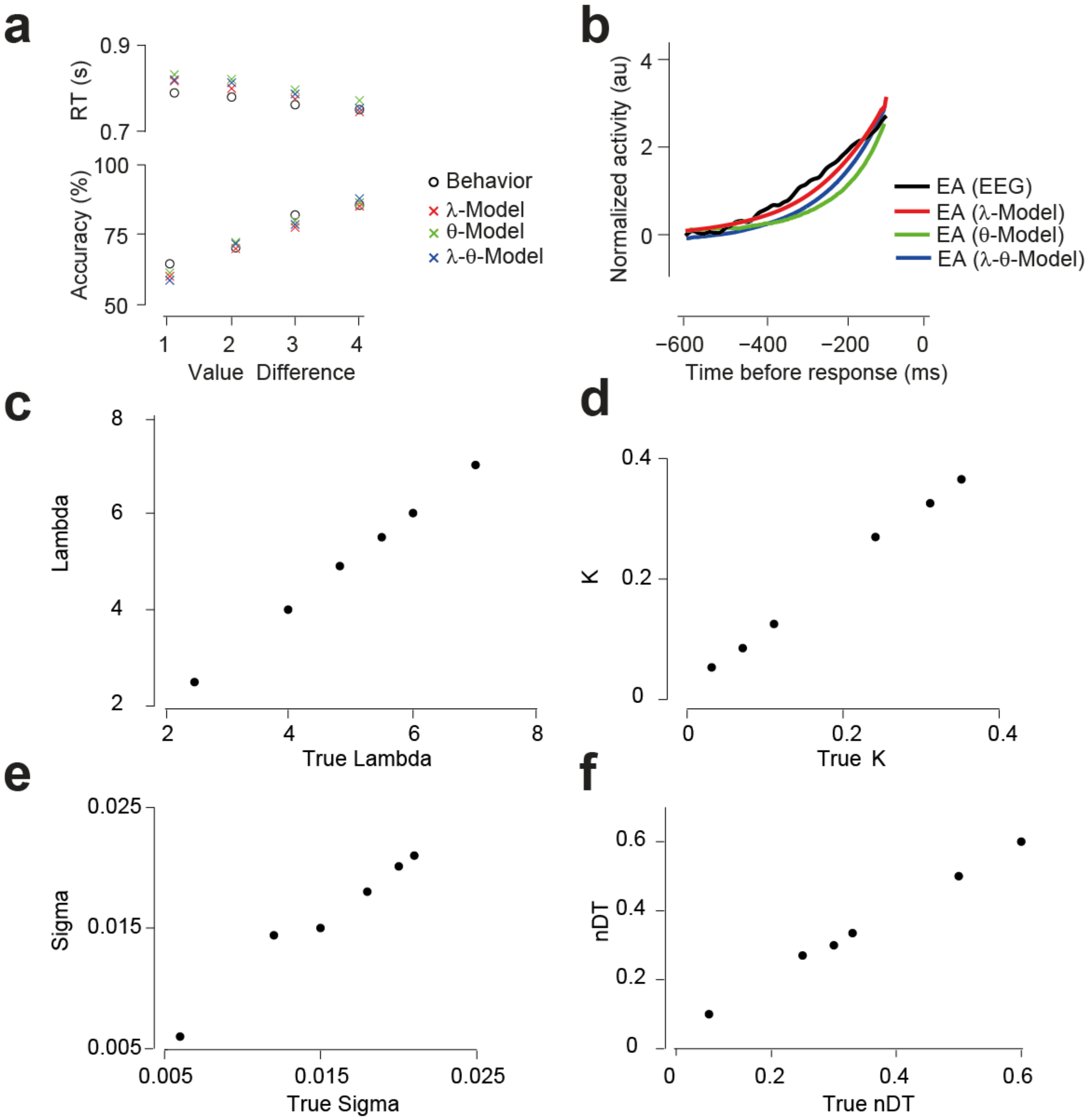

Supplementary Figure 5. Alternative SSM models and parameter recovery analysis (see Supplementary Methods below for details)**.** **(a)** Behavioral performance (red circles) and results from the λ, θ and λ+θ model (black, dark and light gray crosses respectively). All models captured participants' average (*N* = 21) reaction time (RT) and accuracy (top and bottom respectively). **(b)** Average (*N* = 21) predicted evidence accumulation (EA) from λ, θ and λ+θ model (black, dark and light gray respectively) and EEG activity (red) in the time window leading up to the response (on average, 600 to 100 ms prior to the response). **(c-f)** Average λ, k, σ and *nDT* parameters

respectively, recovered for six different simulated data sets. Each dot is the average of 10 fits of the simulated data set. All parameters were recovered reliably – note similarity between simulated (y-axis) and true parameters (x-axis).

SUPPLEMENTARY TABLES

			Peak MNI coordinates (mm)			
**Region**	**Hemisphere**	**BA**	***X***	***y***	***Z***	**Z Value (Peak)**
GLM						
EEG cluster (negative correlation)						
No significant clusters						
EEG clusters (positive correlation)						
Posterior Medial Frontal Cortex	R	24	8	2	40	3.71
Lingual Gyrus	L	18	-20	-72	-2	3.51
Precentral Gyrus	L	6	-44	-14	50	3.34
Value Difference clusters (negative correlation)						
Para Cingulate Cortex	R	32	2	10	54	-3.55
Occipital Fusiform Gyrus	L	19	-26	-64	-10	-3.19
Anterior Insula	L	48	-36	20	8	-2.57
	R	48	36	18	4	-2.57
Value Difference clusters (positive correlation)						
Lateral Prefrontal Cortex	L	46	-32	20	42	3.11
Dorsomedial Prefrontal Cortex	L	10	-10	62	22	2.58
Lateral Orbitofrontal Cortex	R	45	56	36	-2	2.62
	L	45	-50	28	-4	2.44
Dorsal Striatum	L	-	-30	-10	0	3.11
Dorsal Striatum	R	-	30	-2	2	2.72
Amygdala	L	-	-26	-10	-14	3.04
Superior Temporal Gyrus	L	22/42	-62	-46	24	3.36
Angular Gyrus	L	40	-56	-52	44	3.31
RT clusters (negative correlation)						
Dorsomedial Prefrontal Cortex	L	10	-2	62	24	-4.89
Ventromedial Prefrontal Cortex	L	10	-2	46	0	-3.87
Lateral Occipital Cortex (extending to Angular Gyrus)	L	7/19	-40	-74	42	-5.25
Postcentral Gyrus	R	3/4	42	-20	54	-4.84
Middle Temporal Gyrus	L	21	-60	-6	-18	-3.98
-	R	21	60	-4	-14	-3.36
Precentral Gyrus	L	4	-4	-22	54	-3.59
Precuneus Cortex	L	23	-6	-52	36	-2.92
RT clusters (positive correlation)						
Temporal Occipital Fusiform Gyrus (Occipital Pole)	R	37	38	-52	-22	6.48
	L	37	-42	-48	-12	5.97
Lateral Occipital Cortex	L	19	-36	-88	-4	5.74
	R	19	42	-78	-6	5.21
Lingual Gyrus	L/R	18	4	-70	-8	5.79
Anterior Insula (extending to posterior Insula)	R	47/48	44	20	-2	5.37
-	L	47/48	-34	24	4	5.08
Superior Frontal Gyrus	R	6	24	-4	72	5.35
-	L	6	-16	-2	74	5.36
Precentral Gyrus	L	6	-32	-6	62	5.02
Posterior Cingulate Cortex	L	23	-2	-28	30	4.50
Superior Temporal Gyrus	R	21/22	50	-36	6	4.38
Thalamus	L	-	-12	-24	10	4.35
-	R	-	8	-16	10	3.84
Lateral Prefrontal Cortex	R	45/46	46	46	14	4.33
-	L	45/46	-44	42	18	4.50
Brain-Stem	L/R	-	10	-26	-26	4.32

VSTIM clusters (negative correlation)						
Superior Temporal Gyrus	R	40	60	-36	36	5.89
-	L	40	-60	-38	36	6.37
Inferior Frontal Gyrus	R	48	58	18	6	5.31
Precuneus Cortex	R	7/19	10	-82	44	4.6
-	-	5/7	0	-58	66	4.73
Lateral Occipital Cortex	L	19	-38	-80	34	4.92
Lateral Frontal Cortex	R	45/46	48	48	18	4.58
-	L	45/47	-46	36	-4	4.39
Posterior Insula	L	48	-38	-14	-6	4.71
Posterior Cingulate Cortex	R	23	3	-36	40	4.49
Dorsomedial Prefrontal Cortex	R	9/32	2	38	38	4.44
Superior Temporal Gyrus	R	20/48	44	-16	-10	4.29
VSTIM clusters (positive correlation)						
Occipital Pole	L	19	-28	-78	-8	6.13
-	R	19	26	-78	-10	6.34
Posterior Medial Frontal Cortex	L	6	-6	-4	54	3.81
Precentral Gyrus	L	4	-40	-22	56	5.19

Supplementary Table 1. BOLD activations in the four predictors of our GLM. Complete list of activations correlating negatively or positively with the single-trial variability in our EEG-derived predictor, with the parametric Value-Difference predictor, with the parametric Reaction Time (RT) predictor and with the unmodulated regressor for the presentation of the stimuli (VSTIM) (GLM; mixed effects, $|Z| > 2.57$, corrected). MNI, Montreal Neurological Institute; L, left hemisphere; R, right hemisphere, BA, Broadmann Area.

λ	K	σ	nDT
3.5	0.28	0.021	0.40
3.5	0.12	0.012	0.30
5.5	0.16	0.009	0.30
5.5	0.16	0.009	0.30
4.5	0.28	0.024	0.30
3.5	0.28	0.024	0.40
3.5	0.08	0.006	0.10
7.0	0.12	0.009	0.30
6.0	0.28	0.012	0.25
3.5	0.20	0.012	0.20
5.5	0.28	0.021	0.30
4.0	0.12	0.009	0.30
6.0	0.16	0.009	0.25
6.5	0.24	0.015	0.20
4.5	0.20	0.021	0.35
6.0	0.20	0.012	0.35
3.5	0.24	0.018	0.35
5.0	0.24	0.018	0.40
4.0	0.16	0.012	0.20
4.0	0.28	0.015	0.30
3.5	0.16	0.018	0.30
**4.69**	**0.20**	**0.01**	**0.29**

Supplementary Table 2. Parameter estimates of the Sequential Sampling Model fitted to individual subject data. We modeled Evidence Accumulation (EA) through the equation: $EA(t+1) = EA(t) + (\lambda \times EA(t) + k\ VD)dt + N(0, \sigma)$ where *VD* is the Value Difference which drives the accumulation, *k* is a parameter that modulates the input, *λ* is a parameter that denotes the leak strength (or urgency) of

the process and $N(0, \sigma)$ is a Gaussian noise term with standard deviation σ. We accounted for early visual encoding of the stimuli and motor execution by adding a non-decision time *nDT* to the time taken to reach the threshold. Each row corresponds to one of the twenty-one subjects. Last row are averages across subjects.

SUPPLEMENTARY METHODS

Perceptual task and Passive Viewing

Eight additional subjects (4 males, 4 females) were recruited with the same selection criteria of the Value-Based (VB) task to perform two control experiments while EEG data were recorded. Each task consisted of 1 block of 100 trials each where subjects were shown a sequence of pairs of the same snack stimuli used in the VB task. Subjects first completed a passive viewing task during which they were asked to passively view the stimuli and focus on their content. Subsequently participants completed a second, perceptual decision making task using the same pairs of stimuli but this time were asked to indicate which of the snack stimuli was the largest (i.e. occupied more space on the monitor), by pressing the left or right arrow key on the keyboard. In both tasks the stimuli and temporal order of events in a trial were identical to the ones used in the VB task. Before the tasks, participants performed the same eye-movement calibration experiments described in the Methods for the VB task.

EEG data was acquired as described in the Methods for the VB task using the same amplifiers and EEG caps used for the VB task. Similarly, all offline pre-processing stages were identical to those used in the VB task, apart from the gradient and BCG artifact removal steps, which were omitted. We computed EEG stimulus and response-triggered traces for all subjects by averaging together all trials and electrodes in the centroparietal cluster defined in the VB task and shown Figure 1c. The response-locked trace in the Passive Viewing condition was obtained by assuming subjected responded with the same mean RT as in the perceptual decision making task and it is only used for visualisation purposes.

Alternative SSM models

We used three new model parameterizations where we introduced a threshold parameter θ to potentially account for individual subject variability in the decision boundary. In the first model we

substituted the leak parameter λ with a threshold parameter θ ('boundary' model). In the second model we used both the leak and boundary parameters ('joint' model), in addition to the other three parameters we used originally (i.e. k, σ, nDT). Finally, in the third model we only used three parameters (i.e. k, σ, nDT) without using λ or θ ('core' model). Specifically, we ran the new models on the same parameter intervals we used in our original model, while we allowed the threshold parameter θ to vary in the range *[0.5:1.5]*. Overall, the new models did capture the main behavioral effect in terms of RTs and accuracy, but they did not appear to provide a better fit to the data compared to our original leaky accumulator model (Supplementary Figure 5a-b). We formally compared the performance of the two new models against the original one using the Bayesian Information Criterion (BIC) and our leaky accumulator model marginally outperformed the 'boundary', the 'joint' model and the 'core' models (BICs: 696.53, 709.31 and 741.49, 836.42 respectively). Expanding the range for k and σ further in the 'boundary' model failed to further improve the fit.

Model parameter recovery analysis

Following the procedure described in [3] to recover the parameters that instantiate the process of evidence accumulation, we assessed the degree to which we could reliably estimate model parameters given our fitting procedure (i.e. via simulations – see Methods). More specifically, we generated one simulated behavioral data set (i.e. full RT distributions for correct and incorrect trials) by running 5000 simulations of the SSM for each VD level using the average parameters estimated originally on the real behavioral data (i.e. as in Supplementary Table 2). Additionally we generated five more simulated behavioral data sets using five randomly sampled parameter sets from the range used in the original fit. For each simulated behavioral data set we ran the SSM this time trying to fit the RT distributions and identify the set of model parameters that maximized our KS statistic in the same way we did for original behavioral data. To assess the recoverability of our parameters we repeated this procedure 10 times for each simulated data set (i.e. 60 repetitions). The recoverability

of the parameters of our SSM was high in almost all cases as can be seen in Supplementary Figure 5c-f.

Probabilistic reward-based (RB) task

The experiment[4] consisted of 2 blocks of 170 trials each, separated by a break (340 trials in total). Before each block, subjects were shown three symbols (randomly picked from a set of 12 symbols) and asked to identify which one had the highest reward probability. They were also told that in each block, the highest reward probability might shift from one symbol to one of the other two and that they would receive a payment (up to £45) based on the outcome of a random set of trials plus an additional payment for attendance (£15).

Subjects were instructed to focus on the central fixation cross to minimise saccades. After a random delay (1–4 s; mean 2.5 s) two of the three symbols briefly appeared to the left and to the right of the fixation cross. To reduce cognitive load, we presented the three possible pair combinations of the symbols in a fixed order (that is, AB, BC, CA), while the side of the fixation cross where the symbols were presented (left or right) was randomized. Subjects had 1.25 s to choose one of the symbols by pressing the left or right button with their right index or middle finger, respectively. After the choice, the fixation cross flickered for 100 ms. The outcome of the decision was presented after a second random delay (1–4 s; mean 2.5 s). A tick or a cross appeared in the centre of the screen for 650 ms to signal a positive or negative outcome, respectively. If subjects failed to respond within 1.25 s the trial was excluded from further analysis.

At any time during the experiment, one of the three symbols was associated with a ‘high’ reward probability of 0.7 compared with the remaining two had a reward probability of 0.3. Participants were not told the exact reward probabilities and were asked to learn to choose the best symbol on each trial by taking into account the decision-outcomes. A few trials after a certain learning criterion was

reached (subjects chose the good symbol in five out of the last six trials), a reversal was introduced by randomly re-assigning the ‘high’ reward probability to a different symbol.

SUPPLEMENTARY REFERENCES

1. Wyart, V. & Tallon-Baudry, C. How Ongoing Fluctuations in Human Visual Cortex Predict Perceptual Awareness: Baseline Shift versus Decision Bias. *J. Neurosci.* **29,** 8715–8725 (2009).
2. Gherman, S. & Philiastides, M. G. Neural representations of confidence emerge from the process of decision formation during perceptual choices. *Neuroimage* **106,** 134–143 (2015).
3. Miletic, S., Turner, B. M., Forstmann, B. U. & Van, L. Parameter Recovery for the Leaky Competing Accumulator Model. *J. Math. Psychol.* **76,** 25–50 (2016).
4. Fouragnan, E., Retzler, C., Mullinger, K. & Philiastides, M. G. Two spatiotemporally distinct value systems shape reward-based learning in the human brain. *Nat. Commun.* **6,** 8107 (2015).
